# Microstructure Identification of Additive Manufactured Titanium Alloy by Using Lamb Wave-DenseNet Network

**DOI:** 10.3390/s25216630

**Published:** 2025-10-28

**Authors:** Yufeng Huang, Yang Zhao, Gang Zhao, Pinghua Yang

**Affiliations:** 1 School of Information Science and Engineering, Harbin Institute of Technology (Weihai), Weihai 264209, China; huangyf00714@163.com (Y.H.); zg18261945768@163.com (G.Z.); 2 Weihai Key Lab of Photoacoustic Testing and Sensing, Weihai 264209, China; 3 AECC Beijing Institute of Aeronautical Materials, Beijing 100095, China

**Keywords:** additive manufacturing, titanium alloy, microstructure, lamb wave intelligent detection, neural network

## Abstract

In the additive manufacturing (AM) process, dynamic fluctuations in process parameters often result in non-uniform grain sizes in the microstructures of fabricated components, which impairs their stability of mechanical performance. Consequently, the accurate identification of microstructures in AM titanium alloy components is essential for optimizing their mechanical reliability and prolonging their service life in engineering applications. An approach combining ultrasonic testing and deep learning is provided to address the demands for high efficiency and intelligent identification of diverse grain microstructures in AM titanium alloys. First, the Centroidal Voronoi Tessellations (CVT) algorithm was employed to construct three representative simulation models that replicate the characteristic grain microstructures of AM titanium alloys encompassing fine-grained, coarse-grained, and mixed-grained configurations. Subsequently, COMSOL Multiphysics software (v.6.3) was utilized to perform laser-induced ultrasonic Lamb wave (LIULW) testing simulations on the CVT-based microstructure models. Further, a comprehensive simulation dataset was established, including time-domain signals and their frequency-domain features of LIULW. This simulation dataset was then used to train a neural network with an improved architecture, aiming to enhance the discriminative capability for subtle differences in LIULW signals induced by varying grain sizes. Experimental validation results demonstrated that the proposed enhanced Lamb wave-DenseNet network achieved an overall recognition accuracy of 97.93% for the three distinct grain microstructure categories. Collectively, these findings confirm that the integrated method provides a robust theoretical framework and a practical technical solution for large-scale, engineering-level microstructure identification of AM titanium alloy components. This work not only bridges the gap between microstructural simulation and intelligent LIULW testing but also lays a foundation for quality control in high-volume AM of titanium alloy structural parts.

## 1. Introduction

Titanium alloys are widely utilized across diverse sectors, including aerospace [1], biomedical engineering [2], shipbuilding [3], and precision instrumentation [4], due to their exceptional mechanical and chemical properties [5]. Additive manufacturing (AM), with its unique capacity for layer-by-layer fabrication and seamless integration of digital design, facilitates the precise production of complex titanium alloy components. These components demonstrate enhanced mechanical properties and consistent quality [6], thereby advancing modern manufacturing technologies. Nevertheless, dynamic fluctuations in equipment parameters during the AM process can result in heterogeneous grain size distribution within components, leading to diminished stability in mechanical performance [7]. Such variability may compromise safety in critical applications. Consequently, rigorous evaluation of the microstructure in additively manufactured titanium alloy components is imperative to ensure operational reliability and structural integrity.

Nondestructive testing (NDT) technologies are widely adopted across various industries due to their ability to evaluate structural integrity without compromising the performance or functionality of the tested object [8,9,10]. Ultrasonic nondestructive testing, distinguished by its capacity to accurately inspect thick-walled components, effectively overcomes the limitations of conventional detection methods, establishing itself as a cornerstone of NDT applications. In contrast to traditional ultrasonic bulk wave testing, Lamb wave testing offers the advantage of long-range propagation along the component’s surface. This capability enables rapid, large-area scanning, surpassing the constraints of localized detection and significantly enhancing inspection efficiency. Consequently, Lamb wave testing provides a robust technical solution for large-scale nondestructive evaluation of additively manufactured components [11].

An electromagnetic acoustic transducer was developed to facilitate unidirectional, phase-accurate Lamb wave emission and high-sensitivity echo reception for inspected components. Comparative analysis of simulation outcomes and experimental measurements enabled precise reconstruction of the component’s edge geometry, significantly enhancing edge detection accuracy [12]. Additionally, a rapid detection scheme employing an array probe was proposed. This approach utilized a 1 MHz probe operating in the A1 mode with a 44° wedge angle, selected based on the specimen’s material properties. An integrated wedge structure was subsequently designed, and a signal amplitude-based total focusing method, augmented by acceleration techniques, was implemented for imaging. Experimental results confirmed the method’s efficacy in detecting slot-shaped defects with minimum dimensions of 5 mm in length, 0.3 mm in width, and 2 mm in depth [13]. Furthermore, a nondestructive testing technique optimized for additively manufactured components was introduced, capitalizing on the weak attenuation and multimodal dispersion properties of Lamb waves during propagation. This method achieves precise defect localization by leveraging secondary wave arrivals through multiple propagation paths. Both experimental and numerical simulations substantiate that defects as small as 1 mm in additively manufactured components can be reliably detected with a positioning error of less than 10% [14]. Nevertheless, the detection speed and accuracy of conventional inspection methods remain inadequate for meeting the stringent requirements of additive manufacturing evaluation.

With the rapid evolution of artificial intelligence technologies, their integration into target feature recognition for ultrasonic testing has gained significant traction, markedly improving the reliability and objectivity of inspection outcomes [15]. For example, a deep learning model utilizing a fully connected deep neural network was developed, employing raw ultrasonic echo signals with 6000 sampling points as input data. To facilitate supervised training, porosity was classified into six distinct levels, with experimental results demonstrating an average classification accuracy of 93% in porosity grading tasks [16]. To meet the stringent accuracy and generalization demands for inspecting laser additively manufactured components, the U-Net architecture was enhanced through three strategic modifications: integration of Inception modules, incorporation of ConvLSTM layers, and addition of supplementary skip connections. These enhancements not only bolstered multi-scale defect detection capabilities but also effectively addressed the prevalent issue of overfitting in deep learning models [17]. Furthermore, to improve prediction accuracy, a 1D-CNN regression model was optimized by incorporating multiple convolutional layer blocks, enabling hierarchical extraction of multi-scale features from ultrasonic longitudinal wave responses. Practical application revealed a relative error of only 6.24% in grain size estimation for polycrystalline materials, underscoring the model’s exceptional predictive precision [18]. Collectively, these advancements highlight a robust and promising pathway for achieving intelligent ultrasonic nondestructive evaluation of microstructures in additively manufactured components.

This study aims to develop an advanced online intelligent recognition method for characterizing microstructures in titanium alloy additive manufacturing processes. Simulation experiments were conducted to evaluate the efficacy of laser-excited Lamb waves for detecting grain structures in additively manufactured components. By integrating multiplexing techniques and support vector machines (SVM), the Lamb wave-DenseNet model was developed, enhancing feature utilization and improving generalization for small-sample datasets. This research addresses a critical gap in the literature concerning intelligent detection of grain structures in additive manufacturing, offering a novel and effective solution. The paper is structured as follows: Section 2 outlines the theoretical framework, encompassing the construction of a polycrystalline microstructure model for additively manufactured titanium alloys using the Centroidal Voronoi Tessellation (CVT) method, the principles of laser-excited Lamb wave modeling in COMSOL, and the methodology for microstructural signal recognition via the Lamb wave-DenseNet deep-learning approach. Section 3 details the simulation setup, including the COMSOL parameters for laser-excited Lamb wave modeling and the hyperparameters for the deep-learning models. Section 4 presents the simulation results and discusses strategies for intelligent classification and identification of signals corresponding to diverse microstructures.

## 2. Principles

### 2.1. Principles of Simulation Model Establishment

From the perspective of grain microstructure, metallic materials typically exhibit homogeneous polycrystalline structures. However, the application of additive manufacturing (AM) technology often results in a layered grain distribution within the workpiece. Consequently, a two-dimensional model can effectively substitute for a three-dimensional one, enhancing the computational efficiency of simulations for detecting additively manufactured components. In contemporary research, the Centroidal Voronoi Tessellation (CVT) algorithm is widely employed to generate models that simulate the grain microstructure of additively manufactured materials [19].

The CVT algorithm is a special type of Voronoi algorithm. Its core feature lies in the fact that the geometric centroid of each Voronoi region exactly coincides with the seed point that generates that region. This correspondence between the centroid and the seed point endows CVT with unique spatial uniformity and optimization characteristics, greatly enhancing the uniformity of grain spatial distribution. The CVT algorithm can be expressed as in Equation (1).

(1) $$v_{i}=\frac{\int_{V}x\rho (x)dx}{\int_{V}\rho (x)dx}$$

**Figure 1:**
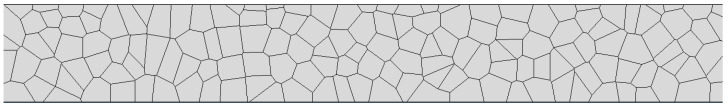
Two-dimensional Voronoi polygon.

In the expression, *v_i_* denotes the centroid of the Voronoi polygon, and *ρ(x)* represents the seed point distribution density function. If *ρ(x)* is constant, the Voronoi polygons are uniformly distributed; otherwise, they are distributed non-uniformly according to the given density field. The case of uniform distribution in two dimensions is illustrated in Figure 1.

Experimental results have shown [20] that this method produces grain structures whose size and shape are closer to the optimal, and it can better reproduce the geometric distribution characteristics of titanium alloy grains.

### 2.2. Principles of Laser-Induced Ultrasonic Lamb Waves Model

Given the advantages of Lamb waves, including their extended propagation distance and capability to detect internal defects, they are well-suited for large-scale detection simulations. During propagation, Lamb waves manifest in two fundamental modes: symmetric and antisymmetric, each characterized by distinct propagation velocities and displacement distributions. In practical applications, the behavior and characteristics of these modes are influenced by several factors, with the frequency-thickness product being paramount. This product governs the excitation and propagation velocities of different modes, playing a critical role in selecting the optimal waveform for ultrasonic inspection.

Lamb waves exhibit multiple modes, and the Rayleigh–Lamb dispersion equation [21] describes the dispersion relationship of Lamb waves propagating in plates with finite thickness. The Lamb waves traveling in a plate-like structure can be decomposed into two fundamental modes, namely the symmetric mode (*S_n_*) and the antisymmetric mode (*A_n_*). These modes correspond to different wave velocities and propagation characteristics, as described by the relationship between wavenumber and frequency-thickness product in Equations (2)–(5).

(2) $$S(n):\frac{\tan(qd)}{\tan(pd)}=-\frac{4k^{2}pq}{{\left(p^{2}-k^{2}\right)}^{2}}$$

(3) $$A(n):\frac{\tan(qd)}{\tan(pd)}=-\frac{{\left(p^{2}-k^{2}\right)}^{2}}{4k^{2}pq}$$

(4) $$p^{2}=\frac{\omega^{2}}{c_{L}^{2}}-k^{2}$$

(5) $$q^{2}=\frac{\omega^{2}}{c_{T}^{2}}-k^{2}$$

**Figure 2:**
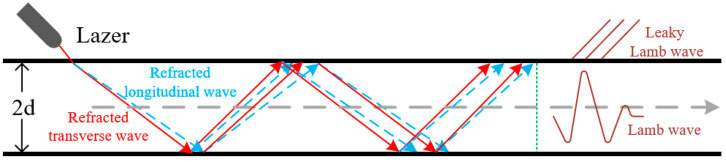
Schematic diagram of LIULW detection.

In the expression, *C_L_* denotes the longitudinal wave velocity of the corresponding mode, *C_T_* represents the transverse wave velocity, *ω* is the angular frequency corresponding to each mode, *d* is half of the plate thickness, and *k* is the wavenumber transmitted in the horizontal direction. In Lamb wave detection, depending on the specific inspection requirements, the appropriate Lamb wave excitation mode should be selected. Different excitation modes correspond to different propagation characteristics, which may be more suitable for detecting different types of defects or evaluating various material properties. Therefore, the excitation frequency range should also be adjusted according to the plate thickness to ensure the excitation of the appropriate Lamb wave mode. A schematic diagram of the detection process is shown in Figure 2.

### 2.3. Neural Network Structure

Convolutional Neural Networks (CNN) represent a cornerstone of deep learning methodologies. Since their inception, CNN have exhibited exceptional adaptability and efficacy in tasks such as two-dimensional image classification and one-dimensional signal recognition, fostering their extensive application across diverse domains. The primary strength of CNN lies in their capacity to transform raw input data into higher-level, semantically rich features through a sequence of concise nonlinear operations. This hierarchical feature extraction facilitates deep representation and precise characterization of the data’s essential attributes. In contrast to traditional machine learning approaches, deep learning technologies, particularly CNN, demonstrate marked improvements in performance as training dataset size increases. This attribute offers a robust technical pathway for extracting valuable insights from the vast datasets generated in the era of information proliferation.

CNN and traditional neural networks share fundamental similarities in their hierarchical architectural design, both leveraging multilayer structures to facilitate progressive information processing and representation learning. However, as an advanced evolution of traditional neural networks, CNN are distinguished by the specialized design and functionality of their constituent layers. Typically, a CNN comprises several core components, including an input layer, convolutional layers, activation function layers, pooling layers, fully connected layers, and an output layer. Furthermore, the architecture of CNN can be flexibly tailored to meet the specific demands of diverse tasks, enhancing their adaptability and performance.

The DenseNet architecture, built upon the foundational principles of CNN, is designed to mitigate challenges such as gradient vanishing, inefficient feature reuse, and parameter redundancy [22]. Building on this framework, the present study introduces the Lamb wave-DenseNet model, specifically developed to differentiate grain microstructures in additively manufactured titanium alloys using one-dimensional signals. The core innovation of this model lies in its direct concatenation of feature maps, where the output of each convolutional layer serves as input to all subsequent layers, establishing a fully connected feature transmission pathway. This explicit feature reuse mechanism enables efficient gradient propagation and information flow, enhancing the model’s performance and robustness.

The convolution module is composed of a convolutional layer, a max-pooling layer, and a Leaky ReLU activation layer. The convolutional layer consists of sixty-four 1 × 5 one-dimensional convolution kernels, which generate thirty-two corresponding feature signals. The operation of the convolutional layer is expressed as in Equation (6), which illustrates how the convolution kernels process the input data to generate feature signals. Through this feature extraction process, we are able to capture meaningful patterns and structures from the raw data, which is crucial for the subsequent model training.

(6) $$f(i)=\mathrm{conv}(\tilde{s},w)=\sum_{i}\tilde{s}(i)w(i)+b$$

In the expression, *w* represents the convolution coefficients, and *b* denotes the bias term. Following the convolutional layer is the activation function layer. In the Lamb wave-DenseNet network, the choice of activation function is crucial as it directly impacts the network’s learning capability and performance. To address the issue of neuron inactivation that can occur with the traditional ReLU function, the Leaky ReLU activation function is chosen in this study. Compared to the standard ReLU activation function, Leaky ReLU allows negative inputs to retain a nonzero slope, meaning that even when the input is less than zero, the neuron does not become completely inactive. This property is especially important for deep neural networks, as it helps prevent the occurrence of “dead neurons” during training, where certain neurons fail to activate due to negative inputs. Its formulation is given in Equation (7).

(7) $$\mathrm{Leaky}\;\mathrm{RELU}(x)=\left\{\begin{array}{ll} x & x\geq 0\\ \alpha x & x<0 \end{array}\right.$$

In the expression, *x* represents the input signal, and *α* is a hyperparameter greater than zero. In the original DenseNet architecture, a fully connected layer is coupled with a softmax classification layer to generate the output. In contrast, this study substitutes the softmax layer with a Support Vector Machine (SVM) to enhance classification accuracy. SVM, a widely adopted algorithm for classification tasks, aims to identify an optimal hyperplane that distinctly separates data points of different classes while maximizing the margin between the hyperplane and the nearest data points, known as support vectors. By leveraging this hyperplane, the feature points generated by the Lamb wave-DenseNet model are accurately assigned to their respective categories. The strength of this approach lies in SVM’s superior ability to manage complex data distributions, particularly in cases with intricate class boundaries. Compared to the global linear decision boundary of the softmax function, the Radial Basis Function (RBF) kernel in SVM provides a nonlinear decision boundary that effectively captures and distinguishes complex, localized differences, thereby achieving higher classification accuracy. SVM enhances classification accuracy by maximizing the margin between classes. The decision hyperplane is formulated as shown in Equation (8).

(8) $$f(x)=\mathrm{sign}\left(\sum_{i=1}^{m}\alpha_{i}y_{i}K(x_{i},x_{j})+b\right)$$

In the expression, *α* denotes the Lagrange multipliers, and *b* represents the calculated decision value. *K(xi,xj)* is the kernel function. In this study, a nonlinear Radial Basis Function (RBF) is employed as the kernel function [23]. The RBF kernel function can effectively handle complex, nonlinearly separable data. In practical applications, by using the RBF kernel, the Support Vector Machine can perform classification more accurately and improve the model’s generalization ability. The formulation of the RBF is given in Equation (9).

(9) $$K(x_{i},x_{j})=\exp\left(-\gamma \|x_{i}-x_{j}{\|}^{2}\right)$$

In the expression, *γ* represents the kernel width control parameter. To prevent the model from overfitting the training data, L2 regularization adds a penalty term of the sum of the squared weights to the loss function, encouraging the model’s weights to remain small. Specifically, L2 regularization works by limiting the model’s complexity, preventing certain features from dominating during training, and thereby improving the model’s generalization ability. Its formulation is given in Equation (10).

(10) $$L_{\mathrm{reg}}(\theta )=L(\theta )+\frac{\lambda }{2}\sum_{i=1}^{n}\theta_{i}^{2}$$

**Figure 3:**
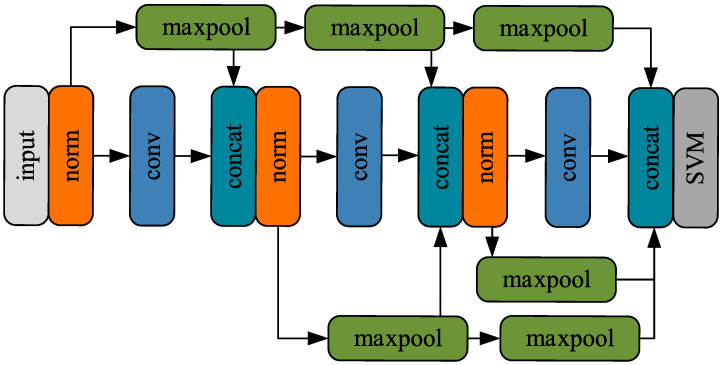
Lamb wave-Densenet network structure.

In the expression, *θ* represents all the parameters of the model, and *λ* is the regularization coefficient. The final architecture of the Lamb wave-DenseNet network is illustrated in Figure 3.

The traditional gradient descent method uses a fixed learning rate, which can lead to uneven convergence speeds at different stages of training or stagnation near local minima. To overcome these issues, this study adopts Adaptive Moment Estimation (Adam) [24] to adaptively adjust the learning rate of each parameter. The update rule is expressed as shown in Equation (11).

(11) $$\theta_{t+1}=\theta_{t}-\frac{\alpha }{\sqrt{{\hat{v}}_{t}}+\epsilon }{\hat{m}}_{t}$$

In the expression, ${\hat{m}}_{t}$ denotes the first-order moment estimate, ${\hat{v}}_{t}$ represents the second-order moment estimate, *α* is the initial learning rate, and *ϵ* epsilon is a small constant introduced to prevent division by zero. *θ_t_* refers to the model parameters at the t-th iteration.

## 3. Simulation Experimental System Setup

### 3.1. Establishment of the Ultrasonic Testing Simulation Model

Given the cost-effectiveness and convenience of simulation experiments, contrasted with the high machining costs and production variability associated with preparing test blocks for physical measurements, this study employs datasets derived from simulation data for neural network training. This approach is utilized to assess the feasibility of leveraging laser-induced ultrasonic Lamb waves (LIULW) for detecting grain microstructures. The efficacy of this methodology, however, is contingent upon the accuracy and reliability of the simulation model.

**Figure 4:**
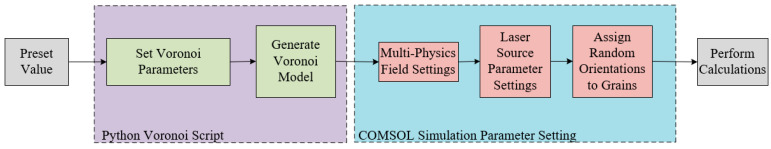
Schematic diagram of the simulation process of grain ultrasonic signal.

The finite element method enables multiphysics coupling and complex model simulations, and has been widely applied in ultrasonic nondestructive testing studies. In this work, Python (v.3.12) scripts are used to construct the grain models, which are then imported into COMSOL software for assigning material parameters and setting the laser ultrasonic parameters. A schematic diagram of the simulation workflow is shown in Figure 4.

This study involves a comparative analysis of four grain microstructure models: the equiaxed grain microstructure (denoted as a), as described in reference [7], typically observed in conventional titanium alloy components, and three microstructures commonly found in additive manufactured components: a mixed microstructure of equiaxed grains and columnar grains (denoted as a + b1), as described in reference [25]; a mixed microstructure of equiaxed grains and anomalous columnar grains (denoted as a + b2), as described in reference [26]; and the columnar grain microstructure (denoted as b), as described in reference [25].

**Figure 5:**
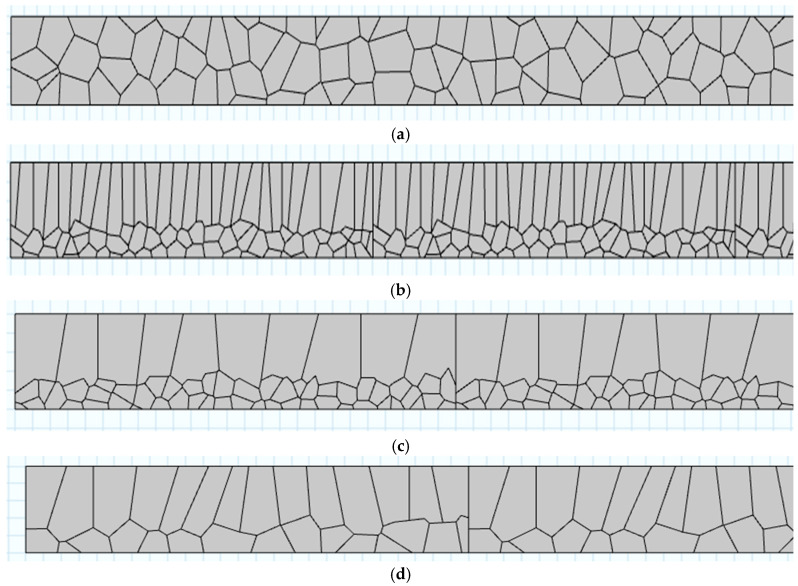
Schematic diagram of titanium alloy grain structure model: (**a**) Equiaxed grains microstructure model; (**b**) Equiaxed grains and columnar grain mixed microstructure model; (**c**) Equiaxed grains and anomalous columnar grain mixed microstructure model; (**d**) Columnar grain microstructure model. All subfigures (**a**–**d**) are synthetic CVT-based simulations, not SEM micrographs. The displayed portion represents a zoomed-in view of the grain structure from the right side of the simulation model, highlighting the microstructural details.

Considering the formation characteristics of Lamb waves, the size of the simulation model in this study is set to 1 mm × 85 mm. Partial schematic diagrams of the four grain microstructure simulation models, constructed based on actual polycrystalline structures, are shown in Figure 5. To clearly display the grain structure, only the 8 mm portion on the right side of the simulation model is shown in the figure.

After importing the model, the simulation structure is constructed as a solid body. The detailed parameter settings are given in Table 1.

Since laser ultrasonic testing involves both the generation of ultrasound waves due to thermal expansion and the ultrasonic detection, the selected physical fields include heat transfer in solids and solid mechanics. In terms of boundary conditions, a heat flux is applied to the upper surface to represent the application of a laser load at a point on the upper surface, while the lower surface is set as a thermally insulated boundary to prevent heat exchange with the external environment. The left and right boundaries are defined as thermally insulated and low-reflection boundaries, respectively, to prevent interference from side reflections on the detection signal. In the multiphysics setup, thermal expansion is selected to couple the two fundamental physical fields. To increase computational accuracy, the mesh is configured as extremely fine.

### 3.2. Deep Learning Parameter Settings

After completing multiple LIULW detection simulations, four sets of simulated detection signals corresponding to different grain microstructures were obtained. To prevent overfitting, Gaussian noise was applied to the signals, resulting in approximately 1320 samples per set, with a total of 5280 samples. Among these, 70% were used as the training set, and 30% were allocated as the validation set. The hardware configuration of the workstation used for deep learning training is shown in Table 2.

To evaluate the effectiveness of the improvements made to the Lamb wave-DenseNet network, this study conducted comparative experiments using the RNN, LSTM, CNN, and DenseNet networks on the same dataset. To ensure scientific rigor in the cross-model comparison, all deep learning models were trained using uniformly configured parameters, in accordance with the standards outlined in Table 3.

**Figure 6:**
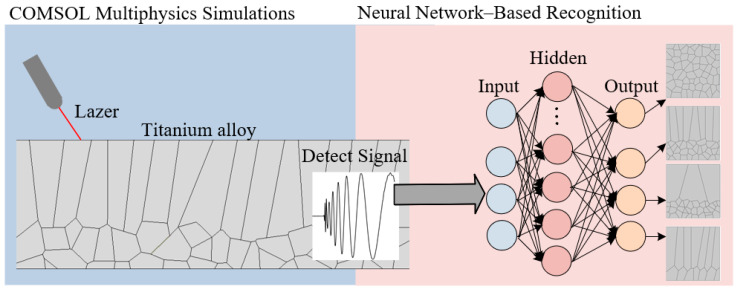
Diagram of intelligent ultrasonic detection system.

With the neural network training parameters configured, all simulation settings are now finalized. The next section presents an analysis of the simulation results. The overall simulation system diagram is shown in Figure 6.

The simulation system consists of two parts: the LIULW detection simulation implemented in COMSOL and the neural network-based intelligent recognition. This section primarily discusses the parameter settings for system setup, while the next section will present a discussion of the experimental results.

## 4. Results and Analysis

### 4.1. Analysis of Simulation Results of LIULW Detection

**Figure 7:**
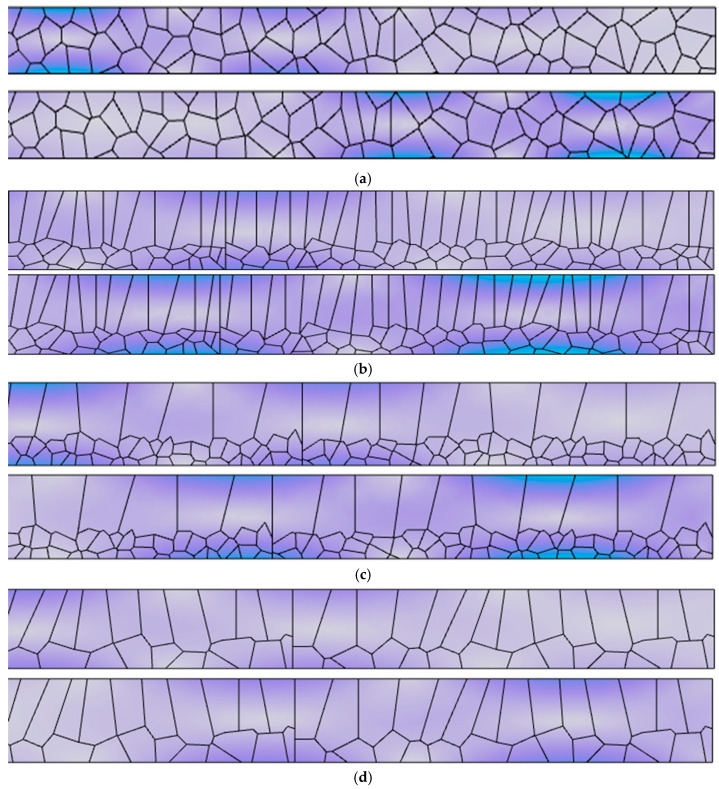
Simulation contour plots of four different Lamb wave grain structure models: (**a**) Simulation contour plot of the equiaxed grain structure model; (**b**) Simulation contour plot of the equiaxed–columnar mixed grain structure model; (**c**) Simulation contour plot of the equiaxed abnormal columnar mixed grain structure model; (**d**) Simulation contour plot of the columnar grain structure model.

The simulation was performed with an output time step of 5 × 10^−8^ s and a total output duration of 1 × 10^−4^ s. Considering the formation characteristics of Lamb waves, the laser excitation source was applied at a position 1 mm to the right of the left endpoint on the upper surface of the model. The simulation contour plots of the four different grain microstructure models at the same time instant are shown in Figure 7. To clearly display the grain structure, only the 8 mm region near the signal receiving position on the rightmost side of the model is shown in the figure.

**Figure 8:**
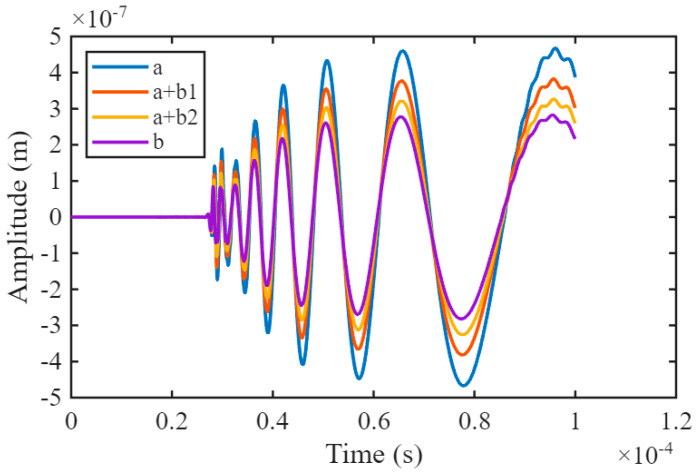
Comparative LIULW signals of the four different grain microstructure models.

From the observation of the simulation contour plots, it can be seen that the propagation process of Lamb waves in the test block during the simulation is consistent with the actual physical phenomenon. This confirms the validity of the LIULW simulation for detecting grain microstructures. Furthermore, a domain point probe is placed 82 mm to the right of the excitation source on the surface of the model to capture the detected simulation signal. The vertical axis represents the vibration amplitude, and the unprocessed waveform is shown in Figure 8.

From the comparison of the simulated LIULW, it can be observed that the greater the proportion of columnar grains in the four different grain microstructure models, the stronger the Lamb wave attenuation. This result is consistent with experimentally verified conclusions [27]. The Lamb wave attenuation differs significantly among the four types of grain microstructure models, confirming the rationality of constructing these distinct models. Furthermore, distinct variations in scattering signals are evident across the different grain structure models, further corroborating the validity of employing diverse grain structure models. However, the waveform differences between the models are difficult to distinguish manually. Therefore, this study introduces deep learning for intelligent detection to enhance the accuracy of the analysis.

### 4.2. Analysis of Neural Network Classification Results

Using the LIULW dataset and applying the unified training configuration specified in Table 2, the recognition accuracy and per-signal inference time for the four baseline networks and the improved Lamb wave-DenseNet are summarized in Table 4.

By comparing the recognition accuracy across different networks, it can be observed that traditional sequential neural networks exhibit significantly lower accuracy than convolutional neural networks. Among them, the RNN network achieved the lowest recognition accuracy at 90.72%, while the LSTM network, improved for long sequence recognition, achieved a recognition accuracy of 91.16%. In contrast, the CNN network, due to its high spatial feature extraction efficiency, achieved a recognition accuracy of 94.07%, while the DenseNet network, due to its feature reuse enhancement, achieved a recognition accuracy of 96.21%. Furthermore, the improved Lamb wave-DenseNet network, benefiting from the strong generalization ability of SVM for small samples, achieved a recognition accuracy of 97.93%, which is 1.72% higher than the original DenseNet network, thus enhancing the reliability of the detection results and validating the effectiveness of the improvements.

**Figure 9:**
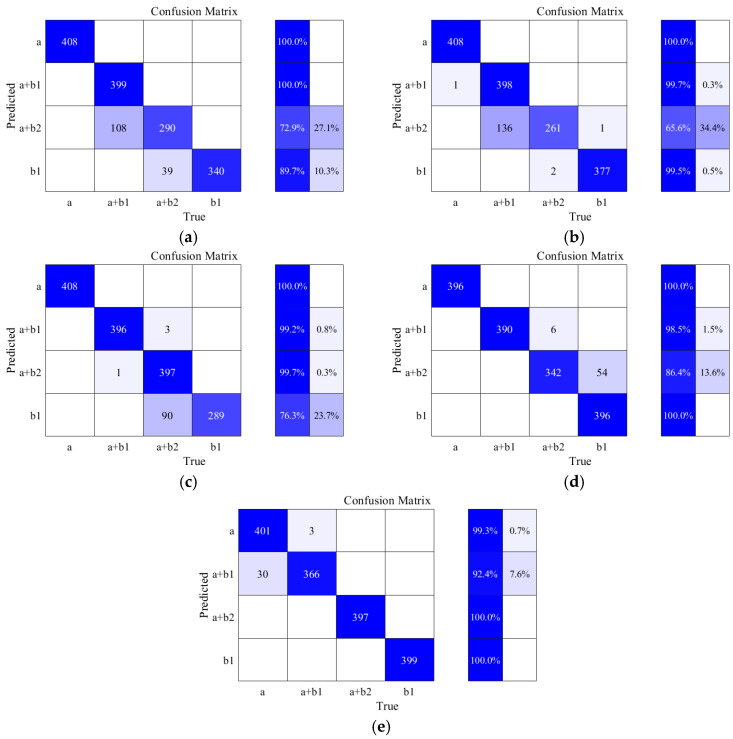
Confusion Matrices of different neural networks: (**a**) Confusion Matrices of RNN; (**b**) Confusion Matrices of LSTM; (**c**) Confusion Matrices of CNN; (**d**) Confusion Matrices of DenseNet; (**e**) Confusion Matrices of Lamb wave-DenseNet.

In terms of recognition time for a single signal, the RNN network achieved the shortest recognition time of 1.57 ms, while the improved Lamb wave-DenseNet network had the longest recognition time of 8.15 ms, which is approximately 2.9 ms longer than the unmodified model. However, in practical detection, the impact of recognition accuracy on detection reliability is prioritized, and the recognition time remains in the millisecond range. Therefore, considering the overall requirements for nondestructive testing performance, the improvements to the network are effective. The confusion matrices of the different models are shown in Figure 9.

From the observation of the confusion matrices, it can be seen that under the LIULW detection dataset, the RNN network shows poor accuracy in distinguishing equiaxed–abnormal columnar mixed microstructures, achieving only 72.9%, and its recognition accuracy for columnar grain microstructures is slightly lower as well. However, its accuracy for the other two microstructures exceeds 90%. The LSTM network demonstrates relatively low recognition accuracy of 65.6% for equiaxed–abnormal columnar mixed microstructures, but achieves over 95% accuracy for the other three models. The CNN network performs poorly in distinguishing columnar grain microstructures, with an accuracy of only 76.3%. The DenseNet network exhibits slightly lower recognition accuracy for equiaxed abnormal columnar mixed microstructures. Meanwhile, the Lamb wave-DenseNet network shows slightly reduced accuracy in identifying equiaxed–columnar mixed microstructures, but performs excellently in recognizing the other three types of grain microstructures. By comparing the confusion matrices of different networks, it is evident that the five neural networks place varying emphasis on the recognition accuracy of Lamb wave signals corresponding to the four grain microstructures, which is attributed to the differences in their network architectures.

Analysis of the recognition outcomes reveals that the enhanced Lamb wave–DenseNet model facilitates intelligent and rapid detection of grain structures in additively manufactured components, while LIULW detection provides non-contact, large-scale, and efficient inspection capabilities. By integrating these methodologies, a real-time monitoring system for additive manufacturing processes can be established. Through the implementation of L2 regularization and the incorporation of Gaussian noise into the dataset, this system effectively mitigates overfitting, enabling precise identification of components with structural inhomogeneities and timely initiation of process interruptions, thereby markedly improving resource utilization efficiency. These findings provide a robust theoretical foundation and practical technical guidance for implementing similar inspection frameworks in real-world production environments. Nevertheless, practical detection must account for challenges such as component surface roughness and the influence of internal micro-defects on signal reflection and attenuation, which may result in discrepancies between experimental and simulation outcomes. These factors also pose challenges for domain adaptation between simulation and experimental data. To enhance the accuracy of simulation results in future applications, the model should be calibrated using actual microstructural data from additively manufactured titanium alloys and corresponding ultrasonic testing data.

## 5. Conclusions

This study introduces an ultrasonic large-area nondestructive testing methodology leveraging the Lamb wave–DenseNet network to simulate the detection of grain microstructures in additively manufactured titanium alloys. During the simulation modeling phase, the Centroidal Voronoi Tessellation (CVT) algorithm was utilized to construct diverse grain microstructure models for these alloys. The interaction between laser-induced ultrasonic Lamb waves and these microstructures was simulated to generate representative detection signal datasets. In the neural network development phase, the conventional DenseNet architecture was refined and extended to create a specialized Lamb wave–DenseNet model tailored for ultrasonic microstructure detection. The model’s generalization capabilities for small datasets were enhanced through the integration of a Support Vector Machine (SVM) classifier. Experimental results demonstrate that this model achieved a recognition accuracy of 97.93% on the laser-induced ultrasonic Lamb wave simulation dataset, surpassing traditional network architectures in both feature extraction efficiency and recognition reliability. Collectively, this study establishes a robust simulation framework, providing a solid theoretical and methodological foundation for future integration with real morphological test data and experimentally derived microstructures. These results affirm the suitability of the proposed simulation-based approach and underscore its potential to advance nondestructive evaluation and quality assurance in additive manufacturing applications.

## Figures and Tables

**Table 1:** COMSOL simulation parameters.

Setting Options	Value
Laser rise time	10 ns
Laser energy density	5 × 10^11^ W/m^2^
Laser spot radius	0.2 mm
Material	Ti-6Al-4V
Elastic modulus	110 GPa
Density	4.43 g/cm^3^
Coefficient of linear thermal expansion	9.5 × 10^−6^/°C

**Table 2:** Hardware conditions of workstations.

Hardware	Disposition
CPU	Intel Xeon Silver 4210R (Intel, Santa Clara, CA, USA)
Number of CPU cores	20
GPU	Nvidia RTX A5000 (Nvidia, Santa Clara, CA, USA)
RAM	24 G

**Table 3:** Neural network training parameter settings.

Setting Options	Value
Epochs	100
Batch-size	64
Shuffle	every-epoch
InitialLearnRate	0.01
LearnRateSchedule	Piecewise
LearnRateDropPeriod	10
L2Regularization	0.001
Optimize Algorithms	adam

**Table 4:** Neural network training results.

Network	Accuracy	Single Signal Recognition Duration
RNN	90.72%	1.57 ± 0.18 ms
LSTM	91.16%	4.31 ± 0.23 ms
CNN	94.07%	3.74 ± 0.21 ms
DenseNet	96.21%	5.21 ± 0.24 ms
Lamb wave-DenseNet	97.93%	8.15 ± 0.27 ms

## Data Availability

The original contributions presented in this study are included in the article. Simulation scripts and representative datasets can be made available upon reasonable request. Further inquiries can be directed to the corresponding author.

## Abbreviations

The following abbreviations are used in this manuscript:

AM	Additive manufacturing
CVT	Centroidal Voronoi Tessellations
LIULW	Laser-induced ultrasonic Lamb wave
NDT	Nondestructive testing
CNN	Convolutional Neural Network
SVM	Support Vector Machine
RBF	Radial Basis Function
